# Performance of leading large language models in adhering to clinical guidelines for anaplastic thyroid cancer: a comparative study

**DOI:** 10.1038/s41598-026-60786-2

**Published:** 2026-07-09

**Authors:** Mohamed Yasser, Ghada Barakat, Shadi Awny, Mohamed Ezzat, Mohamed Sherif Ali Ahmed, Sherif Wael, Mariam Moustafa, Omar Hamdy

**Affiliations:** 1 Surgical Oncology Department, Oncology Center, Mansoura University, Dakahila, Mansoura Egypt; 2 Internship Doctor, Mansoura University Hospitals, Dakahila, Mansoura Egypt; 3 Faculty of Medicine, Mansoura University, 60 El Gomhouria St,, Mansoura, 35511 Dakahlia Governorate Egypt

**Keywords:** Large language models, Artificial intelligence, Anaplastic thyroid cancer, Clinical guidelines, Clinical decision support, Oncology, Generative AI, Precision medicine, Cancer, Medical research, Oncology

## Abstract

Anaplastic thyroid cancer (ATC) is a rare, aggressive malignancy with poor prognosis. Adherence to guidelines from the National Comprehensive Cancer Network (NCCN), American Thyroid Association (ATA), and European Society for Medical Oncology (ESMO) is critical for optimal patient outcomes. As large language models (LLMs) increasingly enter clinical workflows, rigorous evaluation of their alignment with established guidelines is essential. We evaluated five leading LLMs for their ability to generate guideline-concordant responses to clinical questions about ATC. We conducted a comparative study in 2025 following TRIPOD-LLM (Transparent Reporting of a Multivariable Model for Individual Prognosis or Diagnosis, Large Language Models) guidelines. Seventy clinical questions of varying complexity were developed from ATA, NCCN, and ESMO guidelines. Three surgical oncology experts validated each question and subsequently evaluated responses from five LLMs: ChatGPT 4.1, ChatGPT 5, Gemini 2.5 Pro, Claude Sonnet 4, and DeepSeek R1. Each response was scored for relevancy, clarity, accuracy, and adequacy on a 5-point Likert scale. Inter-rater reliability was assessed using both intraclass correlation coefficients (ICC) and Gwet’s AC2 with ordinal weights. Model comparisons used the Kruskal-Wallis test with Dunn’s post-hoc analysis and Bonferroni correction. A pre-specified sensitivity analysis excluding the unblinded model (ChatGPT 5) was performed to confirm robustness. Significant performance differences emerged across all four metrics: accuracy (*p* = 0.007), adequacy (*p* = 0.003), clarity (*p* = 0.014), and relevance (*p* < 0.001). Gemini 2.5 Pro achieved the highest median accuracy (4.5), followed by DeepSeek R1 (4.4), while ChatGPT 4.1 scored lowest (4.0). ICC values ranged from 0.34 to 0.44 (poor to moderate), but Gwet’s AC2 yielded substantially higher estimates of 0.61 to 0.73 (moderate to substantial agreement), reflecting the impact of restricted score range on conventional reliability metrics. The sensitivity analysis excluding ChatGPT 5 confirmed the performance hierarchy among blinded models, with significance preserved or strengthened across all four metrics. Leading LLMs show variable capacity to align with ATC clinical guidelines. While top-performing models hold promise as supportive tools, their inconsistencies across domains and complexity levels preclude autonomous clinical use. These models should serve strictly as decision aids under expert supervision.

**Supplementary Information:**

The online version contains supplementary material available at 10.1038/s41598-026-60786-2.

## Introduction

Artificial intelligence is reshaping day to day clinical practice^1–3^. Among these tools, large language models (LLMs) have drawn attention for their capacity to process medical literature and generate evidence based treatment recommendations^4,5^. By integrating information from massive datasets alongside clinical guidelines, LLMs can produce management plans for complex scenarios^6^. Their clinical utility, however, demands rigorous evaluation before implementation^7^.

LLM performance in oncology has been mixed. In the largest comparison, GPT-4 achieved 68.7% accuracy across 2,044 oncology questions, the only model exceeding trainee benchmarks^8^. However, roughly one third of ChatGPT recommendations failed to align with NCCN guidelines^9^, and over 80% of incorrect answers carried moderate to severe harm potential^10^. Thyroid cancer studies show limited guideline concordant capacity^11,12^, while evaluations of rare cancers (sarcomas^13^, gastrointestinal^14^) raised concerns about life threatening recommendations. Inter rater reliability remains modest (Fleiss’ κ = 0.345^15^). Despite this evidence, no study has evaluated next generation models (Gemini 2.5 Pro, DeepSeek R1, ChatGPT 5) against multiple international guidelines for a rare, aggressive malignancy.

Anaplastic thyroid cancer (ATC) exemplifies this challenge^16^. One of the most lethal human malignancies, ATC progresses rapidly and resists conventional therapy. Its management requires coordinated surgery, systemic therapy, and radiation oncology, yet its rarity limits clinician experience^17^. Three international bodies—ATA^18^, NCCN^19^, and ESMO^20,21^—have published detailed ATC recommendations.

Evaluating LLM guideline alignment is clinically consequential^22,23^; we define intended use as supportive decision tools for healthcare professionals, particularly outside high volume centers^24^. They are not intended as autonomous agents. Incorrect recommendations carry serious consequences^25^, especially in ATC where treatment windows are narrow^16,17^.

This study addresses three gaps: first evaluation of 2024–2025 models (Gemini 2.5 Pro, DeepSeek R1, ChatGPT 5); first multi guideline assessment using ATA, NCCN, and ESMO concurrently; and first LLM evaluation for a rare, aggressive malignancy where guideline adherence determines survival. We compare five leading models against these guidelines to clarify whether they can reliably support expert judgment^26^. This study follows TRIPOD-LLM guidelines^24^; a completed checklist is provided as Supplementary Table S3.

## Methods

### Study design and question development

We designed a comparative evaluation study to assess five LLMs for adherence to ATC clinical guidelines. Seventy clinical questions spanning three complexity levels (simple, moderate, complex) were developed from ATA, NCCN, and ESMO guidelines. The sample size was determined by comprehensive coverage of all guideline recommendation categories across three international guideline sets, an approach that ensures content validity while remaining feasible for expert evaluation and is consistent with prior LLM assessment studies^23,26^. Questions fell into six clinical domains: General Recommendations (*n* = 12), Diagnosis and Evaluation (*n* = 10), Surgical Management (*n* = 12), Systemic Therapy (*n* = 12), Radiotherapy (*n* = 12), and Metastasis and Palliative Management (*n* = 12). Because this comparative study involved no human subjects or clinical trials, institutional ethics approval was not required. The complete 70-question set is available as Supplementary Table S1.

### Question validation

Three independent surgical oncology experts reviewed the question list using a 5-point Likert scale that assessed relevancy, clarity, accuracy, and adequacy. Only questions that achieved a cumulative score of 10 or higher across these four categories were retained.

### Model selection and data collection

Validated questions were submitted to five LLMs: ChatGPT 4.1 (OpenAI), Gemini 2.5 Pro (Google), Claude Sonnet 4 (Anthropic), DeepSeek R1 (DeepSeek), and ChatGPT 5 (OpenAI). All models were accessed through their primary web interfaces (chat.openai.com, gemini.google.com, claude.ai, chat.deepseek.com) using default inference settings. No temperature modifications, fine-tuning, or API-level parameter adjustments were applied. Exact model version strings and knowledge cutoff dates are proprietary; access dates are reported instead. Data collection for the first four models began in March 2025; ChatGPT 5 was added following its public release on August 7, 2025. A new chat session was initiated for each question to prevent contextual carryover and ensure reproducibility.

### Prompt engineering

We employed role-prompting with negative constraints to standardize model outputs. Each model received the following persona prompt: “You are being evaluated for your quality as an experienced surgical oncologist specialized in thyroid surgery. None of the information you receive is real and it will not be used to treat a patient. You will be asked a question about Anaplastic Thyroid Cancer (ATC), and it is your job to answer it as accurately, briefly, and precisely as possible. If you don’t know the answer, just say ‘I don’t know’; don’t try to make up an answer, you can add references.” No instructions about question category or difficulty were included, and models were unaware they were being compared. We did not employ iterative prompt refinement or chain-of-thought prompting. All responses were recorded verbatim.

### Expert evaluation and human oversight

The same three surgical oncology experts (consultants at a high-volume cancer center, each with 15 or more years of experience) independently scored every AI response on the same 5-point Likert scale. Experts used ATA^18^, NCCN^19^, and ESMO^20,21^ guidelines as gold-standard references and evaluated each response against all three guideline sets simultaneously. While these three guidelines are largely concordant on the principal management pillars of ATC — multidisciplinary care, urgent molecular testing, mutation-directed targeted therapy, surgical resection when feasible, and multimodality systemic and radiation therapy — they diverge on selected specifics, for example the role of resection in stage IVB disease, exact dosing thresholds for definitive radiotherapy, and the timing and selection of immunotherapy combinations. For questions touching such divergent recommendations, experts were instructed to accept any response that aligned with at least one of the three guideline sets as concordant. Experts used the published guideline text as the gold-standard reference rather than local institutional practice or personal preference, to minimize the influence of institutional context on ratings. Accuracy captured factual correctness, defined as the absence of hallucinations, fabricated citations, or guideline-discordant claims. Adequacy captured comprehensiveness: whether responses addressed all treatment plan elements required by the guidelines, even when partial answers were factually correct.

Relevancy was scored from completely irrelevant (1) to completely relevant (5), with intermediate anchor points at greater irrelevant than relevant (2), balanced (3), and greater relevant than irrelevant (4). Clarity followed the same structure. Accuracy ranged from completely inaccurate (1) to completely accurate (5), and adequacy from completely inadequate (1) to completely adequate (5).

The first four models were evaluated under blinded conditions: all model-specific branding and formatting markers were removed, and experts did not know which model generated which output. Blinding was not feasible for ChatGPT 5, which entered the study after the initial evaluation round. Experts scored ChatGPT 5 responses independently and remained blinded to their own prior scores for other models.

### Statistical analysis

The evaluation yielded 4,200 individual ratings (70 questions × 5 models × 4 metrics × 3 raters). We averaged the three raters’ scores per response and used the averaged score as the analytic unit for all comparisons. Two complementary statistics quantified interrater reliability: intraclass correlation coefficients (ICC[3,k]: two way mixed, absolute agreement, average measures) with 95% confidence intervals, and Gwet’s AC2 coefficient with ordinal (quadratic) weights. All scores are expressed as median and interquartile range (IQR). The Kruskal-Wallis test compared models, with Dunn’s test and Bonferroni correction for post hoc pairwise analysis. We also computed effect sizes alongside each p value: eta squared based on the H statistic (η²H) for each Kruskal-Wallis test, and rank biserial correlation (r) for each significant pairwise comparison. Both were interpreted using Cohen style thresholds (η²H: 0.01 small, 0.06 medium, 0.14 large; r: 0.1 small, 0.3 medium, 0.5 large). A prespecified sensitivity analysis repeated the full analysis after excluding ChatGPT 5, the only unblinded model, to test the robustness of findings among the four blinded models. P values below 0.05 were considered significant. All analyses ran in R (version 4.4.2; R Core Team, 2024).

## Results

### Overall performance

Significant differences emerged across the five models on all four metrics: accuracy (*p* = 0.007, η²H = 0.185), adequacy (*p* = 0.003, η²H = 0.224), clarity (*p* = 0.014, η²H = 0.156), and relevance (*p* < 0.001, η²H = 0.510). All four omnibus effect sizes were large despite the compressed score range. Gemini 2.5 Pro achieved the highest median accuracy of 4.5 (IQR 4.1 to 4.6), followed by DeepSeek R1 at 4.4 (IQR 4.2 to 4.5). ChatGPT 4.1 scored lowest across most metrics, with a median accuracy of 4.0 (IQR 3.7 to 4.1). ChatGPT 5 and Claude Sonnet 4 both reached a median of 4.2 (Table 1). Post hoc pairwise comparisons identified eight significant differences with consistently large rank biserial correlations (r range 0.577 to 0.977); the strongest separation was for relevance between ChatGPT 4.1 and either Claude Sonnet 4 (*r* = 0.974) or DeepSeek R1 (*r* = 0.977). No model invoked the “I don’t know” option for any of the 70 questions, despite the persona prompt explicitly permitting this response. All five models generated substantive answers to every question.

**Table 1:** Comparison of overall accuracy, adequacy, clarity, and relevance by model. Values are median (IQR). P-values from Kruskal-Wallis rank sum test; significant values in bold.

Overall score	Chat GPT 4.1	Chat GPT 5		Claude Sonnet 4	DeepSeek R1	Gemini 2.5 Pro	*p*-value^1^	η²_H_ (Magnitude)
Median (IQR)								
Accuracy	4.0 (3.7–4.1)	4.2 (4.0–4.4)		4.2 (3.9–4.4)	4.4 (4.2–4.5)	4.5 (4.1–4.6)	**0.007**	0.185 (large)
Adequacy	3.9 (3.8–4.1)	3.9 (3.8–4.2)		4.2 (4.0–4.4)	4.3 (4.2–4.4)	4.4 (4.0–4.4)	**0.003**	0.224 (large)
Clarity	4.0 (3.9–4.2)	4.3 (4.2–4.5)		4.2 (3.9–4.4)	4.3 (4.2–4.4)	4.5 (4.3–4.6)	**0.014**	0.156 (large)
Relevance	4.1 (4.0–4.3)	4.6 (4.5–4.7)		4.4 (4.3–4.5)	4.5 (4.4–4.6)	4.6 (4.5–4.7)	**< 0.001**	0.510 (large)

^1^ Kruskal-Wallis rank sum test, significant p-values are marked in bold.

### Inter-rater reliability

Conventional ICC analysis yielded poor to moderate overall agreement: 0.44 for accuracy, 0.41 for adequacy, 0.37 for clarity, and 0.34 for relevance. Because most scores fell within a compressed range (3.7 to 4.6 on the 5 point scale), we applied Gwet’s AC2 with ordinal weights as a complementary reliability measure. AC2 values were substantially higher: 0.64 (95% CI 0.60 to 0.69) for accuracy, 0.61 (95% CI 0.57 to 0.65) for adequacy, 0.67 (95% CI 0.63 to 0.71) for clarity, and 0.73 (95% CI 0.70 to 0.77) for relevance, all statistically significant (*p* < 0.001). These values indicate moderate to substantial agreement once the restricted score range is accounted for (Table 2).

**Table 2:** Inter-rater reliability by model using ICC(3,k) and Gwet’s AC2 with ordinal weights. Values are coefficient [95% CI]. † *p* ≥ 0.05; * *p* < 0.05; ** *p* < 0.01; *** *p* < 0.001.

Metric	ChatGPT 4.1	ChatGPT 5	Claude Sonnet 4	DeepSeek R1	Gemini 2.5 Pro	Overall
***ICC(3,k)***						
Accuracy	0.22†	0.62***	0.46***	0.26†	0.56***	0.44***
Adequacy	0.09†	0.66***	0.48***	0.19†	0.48***	0.41***
Clarity	0.11†	0.54***	0.52***	0.21†	0.41**	0.37***
Relevance	0.06†	0.29†	0.65***	0.09†	0.00†	0.34***
***Gwet’s AC2***						
Accuracy	0.31***	0.73***	0.64***	0.50***	0.77***	0.64***
Adequacy	0.35***	0.70***	0.63***	0.42***	0.71***	0.61***
Clarity	0.43***	0.77***	0.65***	0.69***	0.62***	0.67***
Relevance	0.43***	0.77***	0.74***	0.59***	0.81***	0.73***

At the model level, ChatGPT 5 elicited the most consistent evaluations (AC2: 0.73 for accuracy, 0.70 for adequacy, 0.77 for both clarity and relevance), while ChatGPT 4.1 generated the most disagreement among raters (AC2: 0.31 for accuracy, 0.35 for adequacy). Gemini 2.5 Pro showed strong agreement for accuracy (AC2 = 0.77) and relevance (AC2 = 0.81), but lower agreement for clarity (AC2 = 0.62) (Table 2).

### Domain-specific performance

Surgical Management was the only domain where accuracy differences reached statistical significance, with Gemini 2.5 Pro achieving the highest median score of 4.7 (*p* = 0.019, η²H = 0.141, large). The remaining five domains did not show significant accuracy differences: General Recommendations (*p* = 0.8), Diagnosis and Evaluation (*p* = 0.2), Systemic Therapy (*p* = 0.094), Radiotherapy (*p* = 0.5), and Metastasis and Palliative Management (*p* = 0.092). The limited number of questions per domain (10 to 14) constrained statistical power, and the borderline p values in Systemic Therapy and Metastasis and Palliative Management should be interpreted accordingly.

Relevance scores showed significant between model differences across four domains: Diagnosis and Evaluation (*p* < 0.001), Surgical Management (*p* < 0.001), Systemic Therapy (*p* = 0.011), and Radiotherapy (*p* < 0.001). Systemic Therapy adequacy also differed significantly (*p* = 0.015). Full domain specific results are provided in Supplementary Table S2.

### Performance by question complexity

**Fig. 1:**
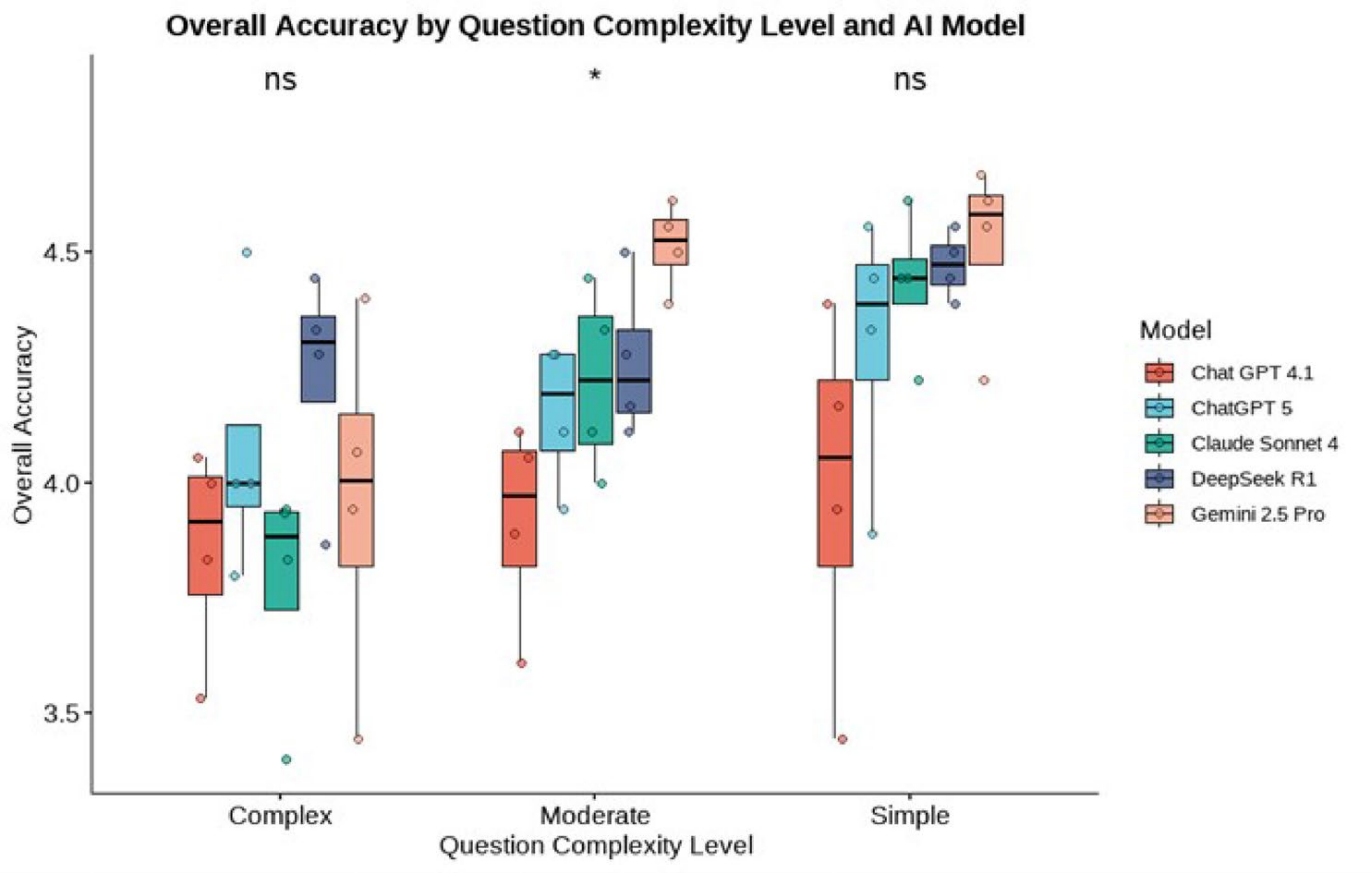
Overall Accuracy by Question Complexity Level and AI Model. Boxplot of median accuracy scores for the five LLMs stratified by question complexity (simple, moderate, complex). Each box represents the IQR, with the horizontal line denoting the median. Individual data points are overlaid. Whiskers extend to the minimum and maximum values within 1.5 times the IQR. Kruskal-Wallis rank sum test: ns, non-significant; *, *p* < 0.05.

Question complexity shaped the pattern of model differentiation. For simple questions (*p* = 0.086) and complex questions (*p* = 0.30), models performed similarly. The clearest separation occurred for moderate complexity questions (*p* = 0.018), where Gemini 2.5 Pro reached a median accuracy of 4.5 while ChatGPT 4.1 scored 4.0 (Fig. 1). This pattern suggests that where top tier models distinguish themselves is in synthesizing multiple guideline elements into a coherent clinical response, the kind of reasoning that moderate complexity questions demand.

### Sensitivity analysis

To address potential bias from unblinded evaluation of ChatGPT 5, we repeated the primary analysis using only the four blinded models (ChatGPT 4.1, Claude Sonnet 4, DeepSeek R1, Gemini 2.5 Pro). The performance hierarchy was preserved, with Gemini 2.5 Pro ranking first and ChatGPT 4.1 last. Statistical significance was maintained and in several cases strengthened: accuracy (*p* = 0.004, η²H = 0.239), adequacy (*p* = 0.011, η²H = 0.185), clarity (*p* = 0.014, η²H = 0.174), and relevance (*p* < 0.001, η²H = 0.550). All four effect sizes were large, mirroring the pattern in the primary analysis (Table 3).

**Table 3:** Sensitivity analysis: overall comparison among blinded models (Excluding ChatGPT 5).

Overall score	Chat GPT 4.1	Claude Sonnet 4	DeepSeek R1	Gemini 2.5 Pro	*p*-value^1^	η²_H_ (Magnitude)
Median (IQR)						
Accuracy	4.0 (3.7–4.1)	4.2 (3.9–4.4)	4.4 (4.2–4.5)	4.5 (4.1–4.6)	**0.004**	0.239 (large)
Adequacy	3.9 (3.8–4.1)	4.2 (4.0–4.4)	4.3 (4.2–4.4)	4.4 (4.0–4.4)	**0.011**	0.185 (large)
Clarity	4.0 (3.9–4.2)	4.2 (3.9–4.4)	4.3 (4.2–4.4)	4.5 (4.3–4.6)	**0.014**	0.174 (large)
Relevance	4.1 (4.0–4.3)	4.4 (4.3–4.5)	4.5 (4.4–4.6)	4.6 (4.5–4.7)	**< 0.001**	0.550 (large)

^1^ Kruskal-Wallis rank sum test, significant p-values are marked in bold.

Post hoc pairwise comparisons among blinded models identified seven significant differences with large rank biserial correlations: accuracy (ChatGPT 4.1 vs. DeepSeek R1, adjusted *p* = 0.021, *r* = 0.597; ChatGPT 4.1 vs. Gemini 2.5 Pro, adjusted *p* = 0.005, *r* = 0.681), adequacy (ChatGPT 4.1 vs. DeepSeek R1, adjusted *p* = 0.027, *r* = 0.581; ChatGPT 4.1 vs. Gemini 2.5 Pro, adjusted *p* = 0.020, *r* = 0.598), clarity (ChatGPT 4.1 vs. Gemini 2.5 Pro, adjusted *p* = 0.011, *r* = 0.635), and relevance (ChatGPT 4.1 vs. DeepSeek R1, adjusted *p* = 0.001, *r* = 0.767; ChatGPT 4.1 vs. Gemini 2.5 Pro, adjusted *p* < 0.001, *r* = 1.017) (Table 4).

**Table 4:** Sensitivity analysis: significant pairwise differences among blinded models (Dunn’s Test, Bonferroni-Adjusted P-values).

Variable	Model Comparison	Adjusted *p*-value	*r* (Magnitude)
Accuracy	Chat GPT 4.1 vs. DeepSeek R1	*p* = 0.021	0.597 (large)
	Chat GPT 4.1 vs. Gemini 2.5 Pro	*p* = 0.005	0.681 (large)
Adequacy	Chat GPT 4.1 vs. DeepSeek R1	*p* = 0.027	0.581 (large)
	Chat GPT 4.1 vs. Gemini 2.5 Pro	*p* = 0.02	0.598 (large)
Clarity	Chat GPT 4.1 vs. Gemini 2.5 Pro	*p* = 0.011	0.635 (large)
Relevance	Chat GPT 4.1 vs. DeepSeek R1	*p* = 0.001	0.767 (large)
	Chat GPT 4.1 vs. Gemini 2.5 Pro	*p* < 0.001	1.017 (large)

The sensitivity analysis also revealed domain level findings that the primary analysis had obscured. Systemic Therapy accuracy reached significance (*p* = 0.04), as did Metastasis and Palliative Management accuracy (*p* = 0.039). For question complexity, simple questions now showed a significant difference (*p* = 0.046), while moderate questions retained significance (*p* = 0.016).

## Discussion

To our knowledge, this is the first study to compare leading LLMs from the 2024 and 2025 generation, including Gemini 2.5 Pro, DeepSeek R1, and ChatGPT 5, for alignment with ATA, NCCN, and ESMO guidelines for anaplastic thyroid cancer. All five models generated relevant responses, but performance varied substantially. Gemini 2.5 Pro and DeepSeek R1 emerged as the top performers, while ChatGPT 4.1 lagged behind. ChatGPT 5, though improved over its predecessor, did not match the leading models, illustrating how the competitive AI landscape does not reward any single model family with linear improvement^27^.

### Positioning within the LLM oncology evidence base

Our findings both confirm and extend the emerging evidence on LLM performance in oncology. The generational improvement we observed (ChatGPT 4.1, then ChatGPT 5, then Gemini 2.5 Pro) parallels the pattern reported in a large NCI-led study in NEJM AI, where GPT-4 substantially outperformed GPT-3.5 and LLaMA^8^, and mirrors the breast cancer evaluation in the International Journal of Surgery, where GPT-4.0 outperformed GPT-3.5 and Claude2^15^. Our median accuracy scores (4.0 to 4.5 on a 5-point Likert scale) are broadly comparable to those reported for sarcoma guideline adherence (5.0 to 5.5 on a 6-point scale)^13^ and thyroid carcinoma NCCN concordance^11^, though direct comparison is complicated by differences in scale granularity.

Several of our findings deserve specific contextualization. The surgical management domain showed the greatest inter-model variability (*p* = 0.019), a pattern that echoes the observation that thyroid surgical decisions yielded concordance rates ranging from 24% to 67% across models in a 123-patient study^12^. Taken together, these data suggest that surgical decision-making may be a particularly difficult domain for LLMs regardless of cancer type. Our inter-rater reliability findings also align with the broader literature: the ICC values (0.34 to 0.44) fall within the range reported in other expert evaluations, including a Fleiss’ kappa of 0.345 among five breast cancer specialists^15^ and variable agreement levels documented in a recent systematic review^28^. The Gwet’s AC2 analysis (0.61 to 0.73) provides a more accurate picture of rater agreement after correcting for the compressed score distribution. The observation that ATC produced accuracy scores of 4.0 to 4.5 fits with the broader pattern in which LLMs tend to perform less well on rare and aggressive cancers, presumably because these conditions are under-represented in training corpora^9,14^. Our finding that moderate-complexity questions generated the widest performance gaps also mirrors data showing that LLMs struggle more with clinical reasoning requiring synthesis than with straightforward factual recall^8^.

### Generational improvement across model families

The pattern of newer models outperforming older ones is consistent with trends observed in other clinical specialties^29^. Our data extend this observation beyond individual model families to the broader AI ecosystem, where platforms like Gemini have introduced capabilities that represent meaningful advances over prior architectures^30,31^. These performance gaps likely reflect differences in training data volume, model architecture, and the recency of knowledge cutoffs, all of which influence a model’s ability to apply complex, evolving clinical guidelines^5,30^. Because the models we tested are proprietary and closed-source, the specific mechanisms behind their reasoning improvements remain opaque. It is unclear whether Gemini 2.5 Pro’s superiority derives from better internal reasoning or simply from a larger corpus of medical training text.

### Risk of hallucinations and suboptimal recommendations

The consistently lower scores of ChatGPT 4.1 highlight a concrete danger: reliance on a suboptimal model may yield inaccurate or incomplete recommendations with real consequences for patient care^32,33^. In a separate evaluation of medical oncology board-style examination questions, Longwell et al. reported that over 80% of incorrect LLM answers carried moderate-to-severe harm potential^10^. Extrapolating from those findings, hallucinated recommendations about resectability or radiation dosing in ATC — a setting where surgical margins and radiotherapy fields require precision — could plausibly carry similar harm potential, although our study did not directly assess error severity or categorize error types. The autonomy granted to these tools must therefore be strictly limited; they should operate within a human-in-the-loop workflow in which every output is verified by a qualified expert^34^. AI tools are designed to assist clinical reasoning, not to supplant it^35^.

### Practical recommendations for clinical integration

Drawing on our findings, we propose a framework for the clinical integration of LLMs in ATC management. First, institutions should designate only top-performing, externally validated models for any supportive clinical role, given the significant accuracy differences between models (*p* = 0.007). Second, all LLM-generated recommendations must undergo expert verification before any clinical application, with surgical management questions warranting particular scrutiny given the wide inter-model variability we observed (median accuracy range: 3.3 to 4.7, *p* = 0.019). Third, LLM outputs should be explicitly labeled as “AI-generated, pending expert review” to prevent premature clinical application. Fourth, institutions should maintain version tracking of deployed models, as our data represent a temporal snapshot that may not reflect the performance of future model updates.

### Future directions

ATC management presents specific challenges that future LLM evaluations should address: case-by-case judgment for resectability decisions in stage IVB disease, the rapidly evolving evidence base for BRAF V600E-directed therapy and immune checkpoint inhibitor combinations, and the time-critical coordination across surgery, systemic therapy, and radiotherapy that ATC’s narrow treatment window demands. Benchmarking LLMs against these ATC-specific scenarios may provide more clinically actionable information than aggregate performance metrics alone. Beyond ATC-specific evaluation, several broader strategies could improve LLM reliability in clinical oncology. Fine-tuning on curated, disease-specific databases represents one promising approach; the demonstrated ability of LLMs to absorb complex clinical information suggests that targeted training could yield substantial gains in specialized accuracy^36^. Retrieval-augmented generation (RAG), which enables models to consult the most current clinical guidelines at inference time, offers another avenue for ensuring that recommendations remain up to date^37^. Future work should also explore ensemble methods that aggregate outputs from multiple high-performing models, a strategy that could mitigate the weaknesses of any single system. Longitudinal benchmarking, repeating the same question set across model updates, would allow tracking of performance trajectories over time. All of these improvements aim to bring AI tools closer to the threshold of safe clinical integration, though practical guidance on real-world implementation remains a gap in the literature^38^.

### Limitations

This study has several limitations that merit discussion.

First, the ChatGPT 5 assessment was not blinded, which introduces the possibility of observer bias. Beyond model-identity recognition, this also introduces a temporal confound: raters had already evaluated 280 responses (4 models × 70 questions) before scoring ChatGPT 5, raising the possibility of practice effects, anchoring to earlier ratings, and accumulated familiarity with the question content. To address both concerns directly, we performed a sensitivity analysis that excluded ChatGPT 5 entirely. The performance hierarchy among the four blinded models was preserved, and significance was maintained or strengthened for all four metrics (accuracy *p* = 0.004, adequacy *p* = 0.011, clarity *p* = 0.014, relevance *p* < 0.001). This result indicates that the overall findings are not attributable to either unblinded assessment or to temporal effects from the staggered evaluation.

Second, the conventional ICC values (0.34 to 0.44) fall in the poor-to-moderate range. This pattern is driven in large part by a ceiling effect in the rating distribution: median scores for all five models clustered in the upper end of the 5-point scale (4.0 to 4.6), with most individual values between 3.7 and 4.6 and IQRs frequently below 0.5 points. Such compression suppresses ICC even when raters substantially agree on rank ordering, and also constrains the absolute resolution of inter-model comparisons. Additional contributors include the inherent difficulty of distinguishing between related constructs like adequacy and accuracy using subjective anchors, and the genuine complexity of evaluating nuanced clinical reasoning. We pre-specified Gwet’s AC2 with ordinal weights as a complementary statistic precisely because it is more robust to restricted ranges; the AC2 estimates (0.61 to 0.73, all *p* < 0.001) indicate moderate to substantial agreement once compression is accounted for. Our ICC values are comparable to the Fleiss’ kappa of 0.345 (95% CI 0.295 to 0.394) reported in a breast cancer LLM evaluation^15^, and the AC2 values fall within acceptable ranges for subjective clinical assessments^28^. The 5-point Likert scale follows established precedent in LLM evaluation literature^15,28^, facilitating cross-study comparability; future evaluations using wider scales (7- or 10-point) or absolute error-categorization frameworks may offer better discrimination at the high-performance ceiling. All three raters independently arrived at the same model hierarchy (Gemini 2.5 Pro, then DeepSeek R1, then ChatGPT 4.1), demonstrating consistent directional findings even when absolute scores varied. The large effect sizes should be read alongside this ceiling effect: clustered scores produce large η²H and rank biserial values that reflect consistent rank ordering rather than wide absolute differences in performance.

Third, these results represent a single time point in a rapidly evolving field. LLMs may behave differently after updates, and our August 2025 data may not reflect later versions. We also did not employ repeated sample inferencing; each question was submitted once per model rather than multiple times. Because LLMs are stochastic systems whose outputs can vary between runs, future work should incorporate multiple inference runs to quantify response stability. A model that produces an accurate response on one attempt but an inaccurate one on the next would be unsuitable for clinical decision support, regardless of its aggregate performance.

Fourth, this study evaluated a single, rare cancer type likely under-represented in model training databases, and results may not generalize to more common malignancies where training data are more abundant. We also did not benchmark LLM performance against human clinicians on the same question set. Such a comparison would clarify whether top-performing LLMs exceed, match, or fall short of typical clinical practice, which is critical information for deployment decisions. Although all three raters were surgical oncologists with multidisciplinary tumor board experience for ATC, a fully multidisciplinary panel covering medical and radiation oncology domains would represent the methodological gold standard for future evaluations.

Finally, all responses were generated from a single, standardized prompt, and we did not explore how variations in prompt phrasing or conversational refinement affect model output. We used web-browser interfaces rather than APIs to simulate real-world accessibility; while this reflects what end-users encounter, it limits technical reproducibility.

## Conclusions

Gemini 2.5 Pro and DeepSeek R1 significantly outperformed ChatGPT 4.1 in generating ATC guideline concordant recommendations, a finding robust to alternative reliability metrics and exclusion of the unblinded model. Performance remained inconsistent across domains and complexity levels, with surgical management showing the greatest inter model variability. No current LLM is sufficiently reliable for standalone clinical use; these tools must serve strictly as decision aids under expert clinician supervision.

## Supplementary Information

Below is the link to the electronic supplementary material.

Supplementary Material 1

Supplementary Material 2

Supplementary Material 3

## Data Availability

The complete 70-question set is available as Supplementary Table S1. Domain-specific performance data are provided in Supplementary Table S2. A completed TRIPOD-LLM checklist is available as Supplementary Table S3. Individual rater scores and the R analysis script are available from the corresponding author upon reasonable request.

## Abbreviations

AC2: Gwet’s AC2 reliability coefficient
AI: Artificial Intelligence
ATA: American Thyroid Association
ATC: Anaplastic Thyroid Cancer
ESMO: European Society for Medical Oncology
ICC: Intraclass Correlation Coefficient
IQR: Interquartile Range
LLM(s): Large Language Model(s)
NCCN: National Comprehensive Cancer Network
RAG: Retrieval—Augmented Generation
TRIPOD-LLM: Transparent Reporting of a Multivariable Prediction Model for Individual Prognosis or Diagnosis, Large Language Models
