# Supplementary material for: Performance of leading large language models in adhering to clinical guidelines for anaplastic thyroid cancer: a comparative study
Source: Sci Rep. 2026 Jul 9;16:21415. doi: 10.1038/s41598-026-60786-2 (PMC13350685; doi:10.1038/s41598-026-60786-2)
Supplement: Supplementary file 2 — Supplementary Material 2 [file 41598_2026_60786_MOESM2_ESM.docx]

**Supplementary Table S3: Completed TRIPOD-LLM Checklist**

*Study: Performance of Leading Large Language Models in Adhering to Clinical Guidelines for Anaplastic Thyroid Cancer*

Research Design: E (LLM Evaluation) | LLM Task: QA (Long-form Question Answering)

*Items marked N/A are not applicable to this research design (E) and task (QA). Page numbers refer to the manuscript.*

| **Section** | **Item** | **Checklist Item** | **Design** | **Task** | **Page / Response** |
| --- | --- | --- | --- | --- | --- |
| **Title** | **1** | Identify the study as developing, fine-tuning, and/or evaluating the performance of an LLM, specifying the task, the target population, and the outcome to be predicted. | All | All | Title page. Title identifies study as evaluating LLM performance (task: clinical guideline adherence), target population (ATC patients), and outcome (guideline-concordant recommendations). |
| **Abstract** | **2** | See TRIPOD-LLM for Abstracts. | All | All | Abstract section. Structured abstract with Background, Methods, Findings, and Interpretation. Reports study design, models evaluated, metrics, key results, and clinical interpretation. |
| **Background** | **3a** | Explain the healthcare context/use case and rationale for developing or evaluating the LLM, including references to existing approaches and models. | All | All | Introduction, paragraphs 1-2. Healthcare context (ATC diagnosis and management), rationale (guideline adherence for patient safety), references to prior LLM oncology evaluation studies. |
|  | **3b** | Describe the target population and the intended use of the LLM in the context of the care pathway, including its intended users in current gold standard practices. | E, H | All | Introduction, paragraph 4. Target population: patients with ATC. Intended use: supportive clinical decision support for healthcare professionals, especially outside high-volume centers. Not autonomous agents. Gold standard: ATA, NCCN, ESMO guidelines. |
| **Objectives** | **4** | Specify the study objectives, including whether the study describes the initial development, fine-tuning, or validation of an LLM (or multiple stages). | All | All | Introduction, paragraph 5. Objective: evaluate (not develop or fine-tune) five LLMs for guideline adherence. Three specific gaps addressed: (1) next-generation models, (2) multi-guideline assessment, (3) rare malignancy evaluation. |
| **Data** | **5a** | Describe the sources of data separately for the training, tuning, and/or evaluation datasets and the rationale for using these data. | All | All | Methods: Study Design. Evaluation dataset: 70 clinical questions derived from ATA, NCCN, and ESMO guidelines for ATC. Training data for the five LLMs are proprietary and not publicly disclosed by their developers; this is noted in Methods: Model Selection. |
|  | **5b** | Describe the relevant data points and provide a quantitative and qualitative description of their distribution and other relevant descriptors of the dataset. | All | All | Methods: Study Design. 70 questions distributed across 6 domains: General Recommendations (n=12), Diagnosis and Evaluation (n=10), Surgical Management (n=12), Systemic Therapy (n=12), Radiotherapy (n=12), Metastasis and Palliative Management (n=12).Three complexity levels (simple, moderate, complex). Full question set in Supplementary Table S1. |
|  | **5c** | Specifically state the date of the oldest and newest item of text used in the development process and in the evaluation datasets. | All | All | Methods: Study Design and Model Selection. Evaluation questions based on ATA 2021 guidelines [11], NCCN Version 2.2025 [10], and ESMO 2019/2022 guidelines [12,13]. LLM training data dates are proprietary and not publicly available. |
|  | **5d** | Describe any data pre-processing and quality checking. | All | All | Methods: Question Validation. Three independent surgical oncology experts validated all questions using a 5-point Likert scale. Questions with cumulative scores >=10 from four categories were selected. |
|  | **5e** | Describe how missing and imbalanced data were handled and provide reasons for omitting any data. | All | All | Not applicable. All 70 validated questions were submitted to all five models; all responses were received and evaluated. No missing data. |
| **Analytical Methods** | **6a** | Report the LLM name, version, and last date of training. | All | All | Methods: Model Selection. Five LLMs: ChatGPT 4.1 (OpenAI), ChatGPT 5 (OpenAI), Gemini 2.5 Pro (Google), Claude Sonnet 4 (Anthropic), DeepSeek R1 (DeepSeek). Exact version strings and training cutoff dates are proprietary; access dates reported (March-August 2025). |
|  | **6b** | Report details of LLM development process, such as LLM architecture, training, fine-tuning procedures, and alignment strategy. | M, D | All | Not applicable (research design E, not M/D). All five models are closed-source commercial products; architecture and training details are proprietary. Referenced developer publications where available [5,6]. |
|  | **6c** | Report details of how text was generated using the LLM, including any prompt engineering and inference settings. | M,D,E | All | Methods: Prompt Engineering. Role-prompting with negative constraints. Full prompt text provided verbatim. Default inference settings via web interfaces; no temperature modifications, fine-tuning, or API adjustments. New chat session per question. No iterative refinement or chain-of-thought prompting. |
|  | **6d** | Specify the initial and post-processed output of the LLM. | All | All | Methods: Prompt Engineering and Expert Evaluation. Initial output: unstructured free-text responses. No post-processing of LLM outputs. Responses recorded verbatim and evaluated by expert panel. |
|  | **6e** | Provide details and rationale for any classification and, if applicable, how probabilities were determined. | All | C, OF | Not applicable (task is QA, not classification or outcome forecasting). |
| **LLM Output** | **7a** | Include metrics that capture the quality of generative outputs, such as consistency, relevance, and accuracy, compared to gold standards. | All | QA | Methods: Expert Evaluation and Statistical Analysis. Four metrics: relevancy, clarity, accuracy, and adequacy, each on a 5-point Likert scale. Gold standard: ATA, NCCN, and ESMO clinical guidelines. ICC for inter-rater reliability. |
|  | **7b** | Report the outcome metrics' relevance to downstream task at deployment time. | E, H | All | Methods: Expert Evaluation. Accuracy (factual correctness, absence of hallucinations) and adequacy (comprehensiveness of guideline coverage) directly relevant to clinical decision support. Discussion: Practical Recommendations for Clinical Integration. |
|  | **7c** | Clearly define the outcome, how the LLM predictions were calculated, the date of inference for closed-source LLMs, and evaluation metrics. | E, H | All | Methods: Expert Evaluation and Statistical Analysis. Outcome: expert-rated guideline concordance on 4 dimensions. Inference via web interfaces. Date: March-August 2025. Metrics: median Likert scores, IQR, Kruskal-Wallis test, Dunn's test with Bonferroni correction, ICC. |
|  | **7d** | If outcome assessment requires subjective interpretation, describe the qualifications of the assessors, any instructions provided, and inter-assessor agreement. | All | All | Methods: Expert Evaluation. Three surgical oncology consultants at a high-volume cancer center with >=15 years experience. Instructions: 5-point Likert scale with defined anchors for each metric (fully specified in Methods). Inter-rater agreement: ICC(3,k) reported in Results and **Table 2** (range 0.34 to 0.44 overall). Gwet's AC2, which accounts for the restricted score range, yielded substantially higher agreement (0.61 to 0.73, all p<0.001; see **Table 2**). |
|  | **7e** | Specify how performance was compared to other LLMs, humans, and other benchmarks or standards. | All | All | Methods: Statistical Analysis. Five LLMs compared head-to-head using Kruskal-Wallis with Dunn's post-hoc test and Bonferroni correction. Gold standard: three international guidelines (ATA, NCCN, ESMO). Human clinician comparison not performed (acknowledged in Limitations). |
| **Annotation** | **8a** | If annotation was done, report how text was labeled, including providing specific annotation guidelines with examples. | All | All | Methods: Expert Evaluation. Each LLM response labeled on 4 dimensions (relevancy, clarity, accuracy, adequacy) using a 5-point Likert scale. Full scale definitions with anchors provided in Methods. |
|  | **8b** | If annotation was done, report how many annotators labeled the dataset(s), the proportion annotated by more than 1 annotator, and the inter-annotator agreement. | All | All | Methods and Results. Three annotators independently evaluated all responses (100% triple-annotated). Inter-annotator agreement: ICC and Gwet's AC2 values are actually reported in **Table 2**. |
|  | **8c** | If annotation was done, provide information on the background and experience of the annotators. | All | All | Methods: Expert Evaluation. Three surgical oncology consultants at a high-volume cancer center (Mansoura University Oncology Center), each with >=15 years of surgical oncology experience. |
| **Prompting** | **9a** | If research involved prompting LLMs, provide details on the processes used during prompt design, curation, and selection. | All | All | Methods: Prompt Engineering. Role-prompting with negative constraints. Full prompt text provided verbatim. Standardized across all five models. No iterative refinement, chain-of-thought, or few-shot examples. |
|  | **9b** | If research involved prompting LLMs, report what data were used to develop the prompts. | All | All | Methods: Prompt Engineering. Prompt designed by the research team based on the clinical evaluation context. No external data used for prompt development. Prompt designed to simulate clinical decision support query. |
| **Summarization** | **10** | Describe any preprocessing of the data before summarization. | All | SS | Not applicable (task is QA, not summarization). |
| **Instruction tuning / Alignment** | **11** | If instruction tuning/alignment strategies were used, what were the instructions, data, and interface used for evaluation? | M, D | All | Not applicable (research design E, not M/D). No instruction tuning or alignment was performed. |
| **Compute** | **12** | Report compute, or proxies thereof, required to carry out methods. | M,D,E | All | All models accessed via free web interfaces with default settings. No computational infrastructure required beyond standard internet access. Inference time not systematically measured. |
| **Ethical Approval** | **13** | Name the institutional research board or ethics committee that approved the study. | All | All | Methods: Study Design. No institutional ethics approval required as this comparative study involved no human subjects, patient data, or clinical trials. |
| **Open Science** | **14a** | Give the source of funding and the role of the funders for the present study. | All | All | Funding section. No funding was received for this study. |
|  | **14b** | Declare any conflicts of interest and financial disclosures for all authors. | All | All | Declaration of Interests. No competing interests declared. |
|  | **14c** | Indicate where the study protocol can be accessed or state that a protocol was not prepared. | H | All | A formal study protocol was not prepared. The methodology is fully described in the Methods section. |
|  | **14d** | Provide registration information for the study. | H | All | This study was not registered in a trial registry. |
|  | **14e** | Provide details of the availability of the study data. | All | All | Data Sharing Statement. The 70-question set, individual rater scores, and R analysis script available from corresponding author upon reasonable request. Supplementary Tables S1-S2 provided. |
|  | **14f** | Provide details of the availability of the code to reproduce the study results. | All | All | Data Sharing Statement. R analysis script available from corresponding author upon reasonable request. |
| **Public Involvement** | **15** | Provide details of any patient and public involvement. | H | All | No patient or public involvement in the design, conduct, or reporting of this study. |
| **Participants** | **16a** | Describe the flow of text/EHR/patient data through the study. | E, H | All | Not applicable. No patient/EHR data were used. 70 questions submitted to 5 models = 350 total responses, all evaluated by 3 experts = 1,050 individual ratings. |
|  | **16b** | Report the characteristics overall and for each data source or setting. | E, H | All | Not applicable (no patient data). Question distribution described in Methods: 6 domains, 3 complexity levels, derived from 3 international guidelines. |
|  | **16c** | Show a comparison of the distribution of important clinical variables. | E, H | All | Not applicable. No clinical patient data involved. |
|  | **16d** | Specify the number of participants and outcome events in each analysis. | E, H | All | Not applicable. No patient participants. Analysis units: 70 questions x 5 models x 4 metrics x 3 raters. |
| **Performance** | **17** | Report LLM performance according to pre-specified metrics and/or human evaluation. | All | All | Results section, Tables 1-4, Figure 1. Performance reported by: overall model comparison, pairwise post-hoc tests, inter-rater reliability, domain-specific analysis, and complexity-level analysis. All pre-specified metrics (accuracy, adequacy, clarity, relevance) reported with medians, IQRs, and p-values. |
| **LLM Updating** | **18** | If applicable, report the results from any LLM updating. | All | All | Not applicable. No model updating was performed. ChatGPT 5 was evaluated as a separate model (added August 2025) rather than as an update to ChatGPT 4.1. |
| **Interpretation** | **19a** | Give an overall interpretation of the main results, including issues of fairness in the context of the objectives and previous studies. | All | All | Discussion, paragraphs 1-2 and 'Positioning Within the LLM Oncology Evidence Base' subsection. Results contextualized against 7 prior studies. Generational improvement, model hierarchy, and competitive landscape discussed. |
| **Limitations** | **19b** | Discuss any limitations of the study and their effects on any biases, statistical uncertainty, and generalizability. | All | All | Limitations section. Five limitations discussed: (1) unblinded assessment of ChatGPT 5, (2) poor-to-moderate ICC values due to restricted score ranges, (3) temporal snapshot and stochastic nature of LLMs without repeated inferencing, (4) focus on a single rare cancer type without human clinician benchmarking, and (5) reliance on a single standardized prompt via web-browser interfaces. |
| **Usability of the LLM in context** | **19c** | Describe any known challenges in using data for the specified task and domain context. | E, H | All | Discussion: Risk of Hallucinations and Limitations paragraphs 4 and 7. Challenges: ATC under-represented in training data, hallucination risk, proprietary model opacity, prompt sensitivity, stochastic outputs, rapidly evolving model landscape. |
|  | **19d** | Define the intended use for the implementation under evaluation, including the intended input, end-user, level of autonomy/human oversight. | E, H | All | Introduction paragraph 4 and Discussion: Practical Recommendations. Intended input: clinical questions about ATC management. End-user: healthcare professionals. Autonomy: strictly supportive, human-in-the-loop only. All outputs require expert verification before clinical application. |
|  | **19e** | Describe how poor quality or unavailable input data should be assessed and handled when implementing the LLM. | E, H | All | Discussion: Risk of Hallucinations and Practical Recommendations. Outputs must be labeled 'AI-generated, pending expert review.' Institutions should designate only validated models. Version tracking required. Not assessed for poor-quality input handling (acknowledged as future work). |
|  | **19f** | Specify whether users will be required to interact in the handling of the input data or use of the LLM, and what level of expertise is required of users. | E, H | All | Discussion: Practical Recommendations. Users (healthcare professionals) must formulate clinical queries and critically evaluate all LLM outputs against current guidelines. Expert-level clinical knowledge required for verification. LLMs should not be used by non-clinical personnel for treatment decisions. |
|  | **19g** | Discuss any next steps for future research, with a specific view to applicability and generalizability of the LLM. | All | All | Discussion: Future Directions. Next steps: (1) fine-tuning on ATC-specific databases, (2) retrieval-augmented generation with current guidelines, (3) ensemble approaches, (4) longitudinal benchmarking across model updates, (5) human clinician comparison, (6) extension to other rare cancers. |

**Abbreviations:** LLM = large language model; E = LLM evaluation; H = LLM evaluation in healthcare settings; M = LLM methods; D = de novo LLM development; QA = long-form question-answering; C = classification; OF = outcome forecasting; SS = summarization and simplification; MT = machine translation; ATC = anaplastic thyroid cancer; ATA = American Thyroid Association; NCCN = National Comprehensive Cancer Network; ESMO = European Society for Medical Oncology; ICC = intraclass correlation coefficient; IQR = interquartile range.
