# Supplementary material for: Performance of leading large language models in adhering to clinical guidelines for anaplastic thyroid cancer: a comparative study
Source: Sci Rep. 2026 Jul 9;16:21415. doi: 10.1038/s41598-026-60786-2 (PMC13350685; doi:10.1038/s41598-026-60786-2)
Supplement: Supplementary file 3 — Supplementary Material 3 [file 41598_2026_60786_MOESM3_ESM.docx]

Table S2: combined Comparison of overall accuracy, adequacy, clarity, relevance by model and question’s domains.

| Score | Chat GPT 4.1 | Chat GPT 5 | Claude Sonnet 4 | DeepSeek R1 | Gemini 2.5 Pro | p-value^1^ | η²_H_ (Magnitude) |
| --- | --- | --- | --- | --- | --- | --- | --- |
| Median (IQR) |  |  |  |  |  |  |  |
| Overall |  |  |  |  |  |  |  |
| Accuracy | 4.0 (3.7-4.1) | 4.2 (4.0-4.4) | 4.2 (3.9-4.4) | 4.4 (4.2-4.5) | 4.5 (4.1-4.6) | **0.007** | 0.185 (large) |
| Adequacy | 3.9 (3.8-4.1) | 3.9 (3.8-4.2) | 4.2 (4.0-4.4) | 4.3 (4.2-4.4) | 4.4 (4.0-4.4) | **0.003** | 0.224 (large) |
| Clarity | 4.0 (3.9-4.2) | 4.3 (4.2-4.5) | 4.2 (3.9-4.4) | 4.3 (4.2-4.4) | 4.5 (4.3-4.6) | **0.014** | 0.156 (large) |
| Relevance | 4.1 (4.0-4.3) | 4.6 (4.5-4.7) | 4.4 (4.3-4.5) | 4.5 (4.4-4.6) | 4.6 (4.5-4.7) | **<0.001** | 0.510 (large) |
| General Recommendations |  |  |  |  |  |  |  |
| Accuracy | 4.0 (4.0-4.3) | 4.3 (4.0-4.5) | 4.3 (3.5-4.5) | 4.0 (4.0-4.5) | 4.3 (4.0-4.3) | 0.8 | -0.048 (small) |
| Adequacy | 4.2 (4.0-4.3) | 4.3 (4.0-4.3) | 4.3 (3.3-4.7) | 4.3 (4.0-4.3) | 4.3 (3.7-4.3) | >0.9 | -0.055 (small) |
| Diagnosis & Evaluation |  |  |  |  |  |  |  |
| Accuracy | 3.8 (3.3-4.3) | 4.5 (4.3-4.7) | 4.3 (4.3-4.7) | 4.2 (4.0-4.7) | 4.3 (4.0-4.7) | 0.2 | 0.047 (small) |
| Adequacy | 3.7 (3.7-4.3) | 4.0 (3.7-4.3) | 4.3 (4.3-4.7) | 4.3 (4.0-4.3) | 4.3 (4.0-4.7) | 0.081 | 0.078 (moderate) |
| Surgical Management |  |  |  |  |  |  |  |
| Accuracy | 3.3 (3.0-4.3) | 3.7 (3.2-4.2) | 3.8 (3.3- 4.5) | 4.2 (4.0-4.5) | 4.7 (4.0-4.7) | **0.019** | 0.141 (large) |
| Adequacy | 3.5 (3.2-4.3) | 3.5 (3.0-4.0) | 4.0 (3.8-4.2) | 4.2 (3.8-4.3) | 4.3 (4.0-4.3) | 0.12 | 0.061 (moderate) |
| Systemic Therapy |  |  |  |  |  |  |  |
| Accuracy | 3.8 (3.3-4.3) | 4.2 (3.5-4.3) | 4.0 (4.0-4.3) | 4.3 (4.0-4.3) | 4.5 (4.2-4.7) | 0.094 | 0.071 (moderate) |
| Adequacy | 3.8 (3.7-4.3) | 3.8 (2.8-4.3) | 4.0 (4.0-4.3) | 4.0 (4.0-4.3) | 4.3 (4.2-4.7) | 0.088 | 0.075 (moderate) |
| Radiotherapy |  |  |  |  |  |  |  |
| Accuracy | 4.0 (3.3-4.7) | 4.5 (4.2-4.7) | 4.3 (3.8-4.7) | 4.5 (4.0-4.7) | 4.3 (4.2-4.7) | 0.5 | -0.017 (small) |
| Adequacy | 4.3 (3.5-4.3) | 4.3 (4.0-4.7) | 4.2 (3.8-4.5) | 4.3 (4.2-4.7) | 4.3 (4.0-4.7) | 0.5 | -0.014 (small) |
| Metastasis & Palliative |  |  |  |  |  |  |  |
| Accuracy | 4.2 (3.8–4.7) | 4.7 (4.2-5.0) | 4.3 (4.0–4.8) | 4.7 (4.3–5.0) | 4.7 (4.5–5.0) | 0.092 | 0.073 (moderate) |
| Adequacy | 4.2 (3.7–4.7) | 4.3 (4.2-4.8) | 4.3 (4.2–4.7) | 4.7 (4.7–4.7) | 4.7 (4.3–4.7) | 0.13 | 0.057 (small) |
| *^1^ Kruskal-Wallis rank sum test, significant p-values are marked in bold.* | | | | | | |  |
